# Global phylogeographical distribution of *Gloeoporus dichrous*

**DOI:** 10.1371/journal.pone.0288498

**Published:** 2023-07-13

**Authors:** Yoonhee Cho, Chang Wan Seo, Paul Eunil Jung, Young Woon Lim

**Affiliations:** School of Biological Sciences and Institute of Microbiology, Seoul National University, Seoul, Republic of Korea; University of Iceland, ICELAND

## Abstract

Phylogeographic analyses are efficient in ecological and evolutionary studies to discover the origin of a lineage, its dispersal routes, and the divergence of ancestral traits. Studies on widespread wood-decay fungi have revealed the phylogenetic division of several polypores based on geographical distribution. In this study, specimens of *Gloeoporus dichrous*, a cosmopolitan polypore species, were collected globally and analyzed for their geographic distribution. Multi-marker Bayesian molecular clock and haplotype analyses revealed a clear division of *G*. *dichrous* populations by continent. The species diverged from its neighboring clades 10.3 (16.0–5.6) million years ago, with Asian and North American populations at the center of divergence. Possible dispersal mechanisms and pathways are predicted and discussed based on the evaluated transfer routes. The biogeography of *G*. *dichrous* analyzed in this study represents a fraction of the polypore evolution and may advance the understanding of the overall evolution of wood-decay fungi.

## Introduction

Understanding the biogeography of an organism allows prediction of evolutionary processes [1], such as speciation [2], dispersion [3], and natural selection [4]. Biogeography is essential for establishing species conservation strategies in anticipation of rapid changes in climate [5] and pathogens [6]. Therefore, studying biogeography is invaluable for estimating and bridging past and future distributions of species. However, fungi are very underestimated in their biogeography compared to animals and plants despite their vast geographic distribution and significant roles as decomposers and symbionts in the ecosystem [7]. Wood decay fungi (WDF) are among the many fungal groups that are difficult to investigate. WDF often have an insignificant and indifferentiable macromorphology [8] that is susceptible to environmental changes [9, 10] and a wide range of micromorphological characteristics that overlap among taxa [11, 12].

Multi-marker phylogenetic analyses are heavily relied upon in various fields [13] to study WDF because they provide high resolution for the classification and differentiation of WDF taxa. Several studies on the biogeographical distribution of WDF have used multifaceted approaches to multi-marker phylogenetic analyses and have revealed geographically dividing WDF groups [14, 15]. For instance, research on the phylogeographical distribution of the wood decay polypore *Meruliopsis taxicola* (syn. *Gloeoporus taxicola*) has revealed a polyphyletic biogeographical pattern within a limited region of Norway [16]. Similarly, a study on the phylogeographic relationship of the *Ganoderma applanatum*-*australe* species complex revealed mating groups that divided into geographical clades [15]. Research on the biogeography of *Laetiporus*, a cosmopolitan polypore, has revealed the origin of the genus and its dispersal routes to the rest of the world [17].

This study investigated whether another global polypore species, *Gloeoporus dichrous* (≡ *Vitreoporus dichrous* [18], which was assessed as *Gloeoporus* in this study, as other monophyletic *Gloeoporus* species have not been revised to *Vitreoporus*), exhibits a phylogenetic biogeographical distribution similar to that of other wood decay polypores. The phylogeographic patterns of *G*. *dichrous* from different parts of the world were analyzed in this study. This study also traced the chronological biogeographical dispersion pattern of the species through molecular dating using Bayesian phylogenetic analysis to estimate the possible ancestral location, speciation period, and dispersal routes of *G*. *dichrous* to the rest of the world. Several mechanisms of the dispersion of *G*. *dichrous* were suggested. The results of this study may improve the knowledge of the divergence and evolutionary processes of WDF.

## Results

### Divergence time and biogeographic diversification

**Fig 1:**
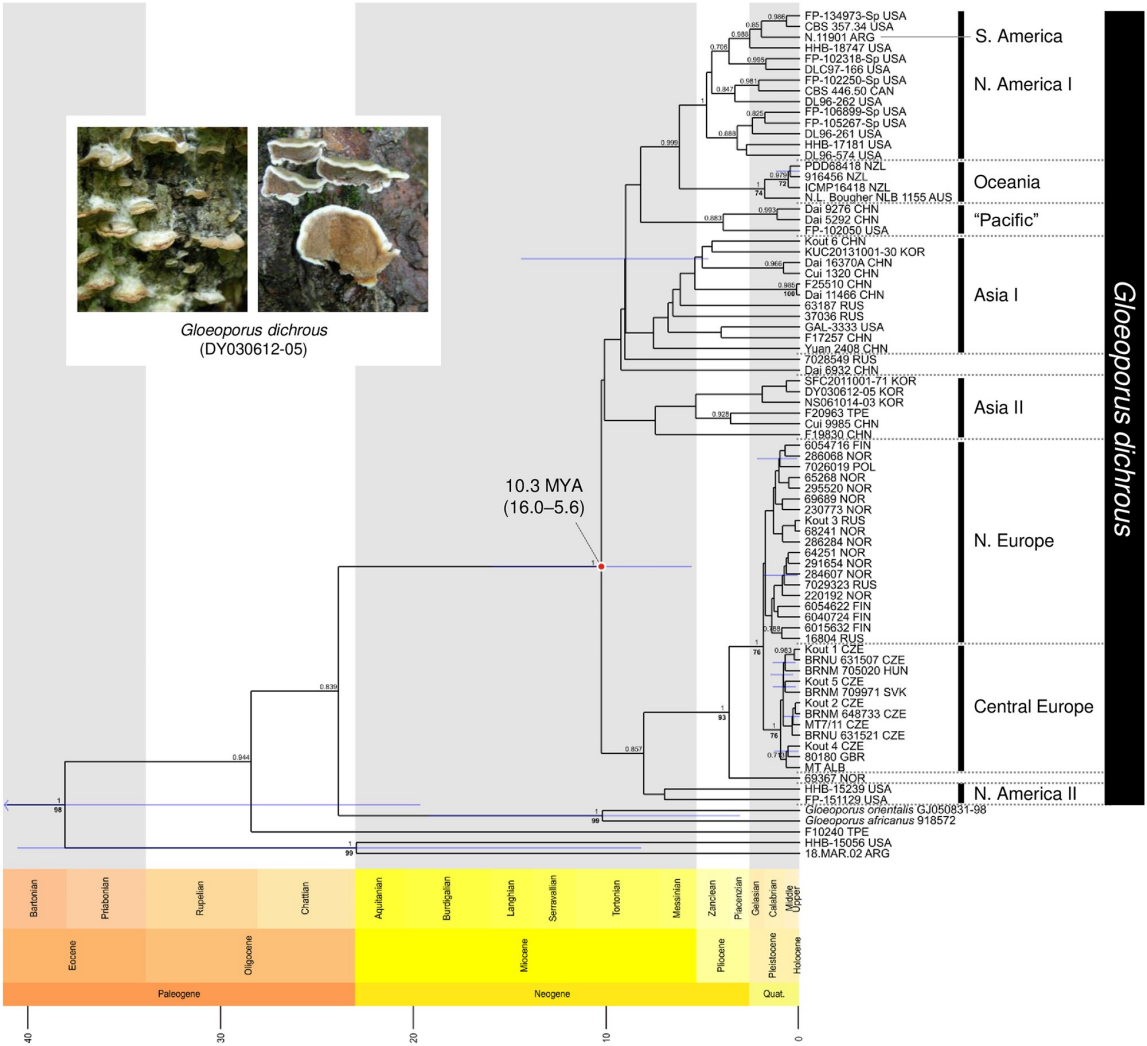
Chronogram for *Gloeoporus dichrous* based on ITS + nrLSU + *rpb2* + *tef1* dataset, constructed using BEAST CladeAge. Only the *G*. *dichrous* clade is shown for clear visualization. The full chronogram can be found in S1 Fig. A geologic timeline and node bars for the highest-posterior-density interval containing 95% of the posterior distribution are displayed. Bootstrap values of ≥ 70 and posterior probability values of ≥ 0.7 are shown. The divergence time of *G*. *dichrous* is indicated by a red circle, and photographs of the fruiting body of *G*. *dichrous* specimen DY030612-05 are provided on the upper left (credit: Y. W. Lim; printed under CC BY 4.0).

Bayesian evolutionary analysis of four genetic regions: Internal transcribed spacer (ITS), nuclear large subunit ribosomal RNA (nrLSU), RNA polymerase II gene (*rpb2*), and elongation factor 1–alpha gene (*tef1*), using BEAST, estimated the population divergence of *G*. *dichrous* as 10.3 (16.0–5.6) million years ago (MYA; Fig 1). *Gloeoporus dichrous* specimens F10240 (Taiwan), HHB-15056 (USA), and 18.MAR.02 (Argentina) were excluded from the monophyletic *G*. *dichrous* clade. The genus *Gloeoporus* diverged 61.0 MYA (median time; S1 Fig). *Gloeoporus africanus*, *G*. *dichrous*, *G*. *orientalis*, *G*. *pannocinctus*, and *G*. *thelephoroides* were grouped within the *Gloeoporus* clade, whereas *G*. *guerreroanus* was grouped within the *Meruliopsis* clade. The family Irpicaceae diverged 79.5 MYA (median time), and the order Polyporales diverged 145.4 MYA (median time). *Gloeoporus phlebophorus* (voucher PDD:105690) grouped distinctly from the Polyporales clade.

**Fig 2:**
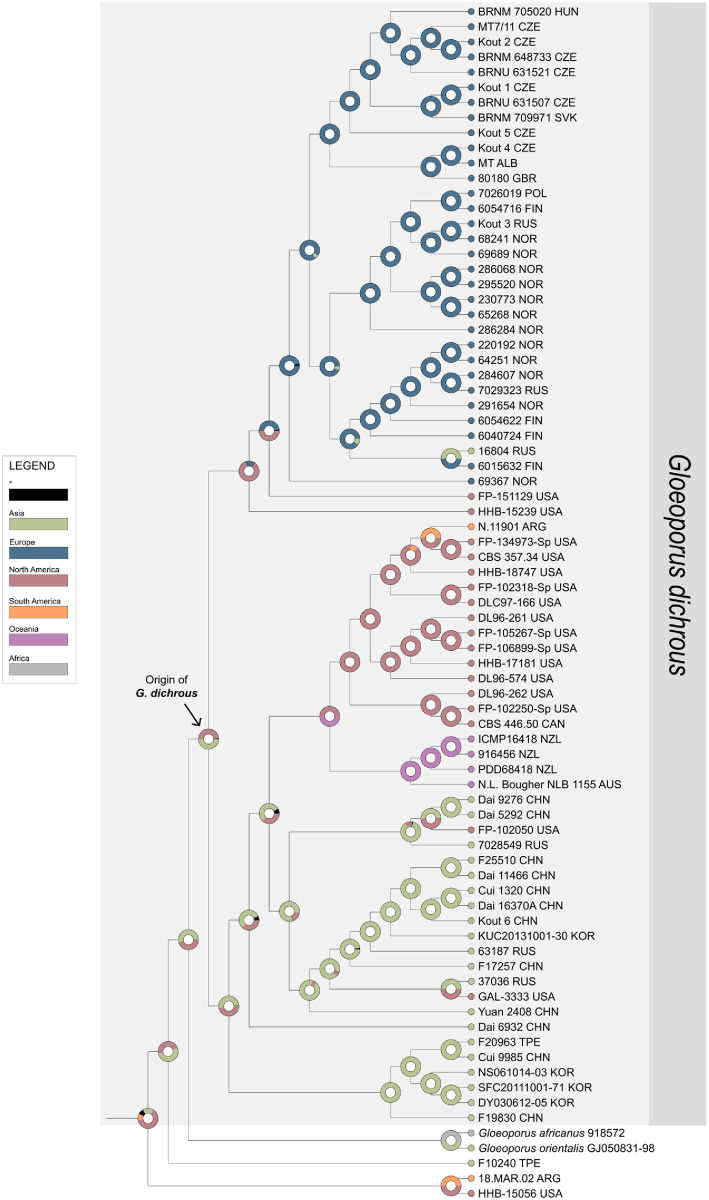
Ancestral area reconstruction of *Gloeoporus dichrous* assessed using the divergence estimation from BEAST. Only the topology is shown for the tree. At each node, the pie chart displays possible distributions inferred from the statistical-dispersal-extinction-cladogenesis (S-DEC) analysis. The outgroup includes *G*. *africanus* and *G*. *orientalis*. Legend for each color is provided on the left. The black asterisk indicates other ancestral ranges.

*Gloeoporus dichrous* specimens were divided primarily into two groups (Figs 1 and 2). One group consisted of clades of Asian specimens, cross-continental specimens (labeled “Pacific”), Oceanian specimens, and North and South American specimens (Fig 1). The Asian clades were further divided into two (labeled I and II), where Asia II clade diverged first from the rest, followed by two single lineages (specimen Dai 6932 from China and specimen 7028549 from Russia), Asia I clade, and then “Pacific” clade. The “Pacific” clade included specimens from China and the USA. The Oceania clade diverged from the remaining North and South American clades. A single specimen from South America was included in North America I clade. The second group consisted of clades comprising European and North American specimens (Fig 1). Specimens from the European group first diverged from the North America II clade, followed by a single lineage (specimen 69367) from Norway. The remaining European specimens were divided into clades of Northern and Central Europe. The Northern European clade included specimens from Finland and Norway, while the Central European clade included specimens from the Czech Republic and Hungary. Specimens from Russia were found in both Asian and European clades. Western Russian specimens were grouped in the Northern European clade, and eastern Russian specimens were grouped with the Asian specimens (Table 1).

**Table 1:** Collection information, haplotype group, and GenBank accession numbers of *Gloeoporus dichrous* specimens.

Specimen	Continent	Country	Location	Host	Haplotype	GenBank accession			
						ITS	nrLSU	*rpb2*	*tef*
Cui 1320	Asia	China	Huangshan, Anhui	Angiosperm	Hap4	**OP295128**	**OP295255**	**OP352336**	**OP352397**
Cui 9985	Asia	China	Antu, Jilin	Angiosperm	Hap4	**OP295129**	**OP295256**	**OP352337**	**OP352398**
Dai 11466	Asia	China	Beijing	*Amydrium* sp.	Hap4	**OP295130**	**OP295257**	**OP352338**	**OP352399**
Dai 16370A	Asia	China			Hap5	KU360399	KU360406		
Dai 5292	Asia	China	Nanjing, Jiangsu	Angiosperm	Hap8	**OP295131**	**OP295258**	**OP352339**	**OP352400**
Dai 6932	Asia	China	Shengyang, Liaoning	*Pinus* sp.	Hap4	**OP295132**	**OP295259**	**OP352340**	
Dai 9276	Asia	China			Hap8	KU360398	KU360407		
F17257	Asia	China	Heilongjiang		Hap4	**OP295133**	**OP295260**	**OP352341**	**OP352401**
F19830	Asia	China	Inner Mongolia		Hap4	**OP295134**	**OP295261**	**OP352342**	**OP352402**
F25510	Asia	China	Beijing		Hap4	**OP295135**	**OP295262**	**OP352343**	**OP352403**
Kout 6	Asia	China	Sichuan		Hap4	**OP295136**	**OP295263**	**OP352344**	**OP352404**
Yuan 2408	Asia	China	Qinshui, Shanxi	*Betula* sp.	Hap4	**OP295137**	**OP295264**	**OP352345**	
DY030612-05	Asia	Korea	Jeollabuk-do	*Pinus densiflora*	Hap4	**OP295138**	**OP295265**	**OP352346**	
KUC20131001-30	Asia	Korea	Gangwon-do	*Abies holophylla*	Hap4	KJ668541	KJ668394		
NS061014-03	Asia	Korea	Gangwon-do		Hap5	**OP295139**	**OP295266**	**OP352347**	**OP352405**
SFC20111001-71	Asia	Korea	Gangwon-do	*Pinus densiflora*	Hap4	**OP295140**	**OP295267**	MG593279	**OP352406**
16804	Asia	Russia	Tyumenskaya oblast		Hap3	**OP295146**	**OP295273**	**OP352351**	**OP352411**
37036	Asia	Russia	Kamchatka, Esso		Hap6	**OP295141**	**OP295268**		**OP352407**
63187	Asia	Russia	Primorskiy krai		Hap4	**OP295142**	**OP295269**	**OP352348**	**OP352408**
7028549	Asia	Russia	Verkhnebureinsky	*Betula lanata*	Hap7	**OP295143**	**OP295270**	**OP352349**	**OP352409**
F10240	Asia	Taiwan	Nantou		Hap5	**OP295144**	**OP295271**		**OP352410**
F20963	Asia	Taiwan	Nantou		Hap4	**OP295145**	**OP295272**	**OP352350**	
MT ALB	Europe	Albania	Vlorë	*Abies borisii-regis*	Hap3	**OP295149**	**OP295276**	**OP352354**	**OP352414**
BRNM 648733	Europe	Czech Republic	Břeclav	*Salix* sp.	Hap3	**OP295150**	**OP295277**	**OP352355**	**OP352415**
BRNU 631507	Europe	Czech Republic	Tábor	*Frangula alnus*	Hap3	**OP295151**	**OP295278**	MG593280	**OP352416**
BRNU 631521	Europe	Czech Republic	Brno	*Alnus glutinosa*	Hap3	**OP295152**	**OP295279**	**OP352356**	**OP352417**
Kout 1	Europe	Czech Republic	South Bohemia	*Salix* sp.	Hap3	**OP295153**	**OP295280**	**OP352357**	**OP352418**
Kout 2	Europe	Czech Republic	South Bohemia	*Alnus* sp.	Hap3	**OP295154**	**OP295281**	**OP352358**	**OP352419**
Kout 4	Europe	Czech Republic	Klatovy	Hardwood	Hap1	**OP295155**	**OP295282**	**OP352359**	**OP352420**
Kout 5	Europe	Czech Republic	Nymburk	Hardwood	Hap3	**OP295156**	**OP295283**	**OP352360**	**OP352421**
MT7/11	Europe	Czech Republic	Břeclav	*Populus* sp.	Hap3	**OP295157**	**OP295284**	**OP352361**	**OP352422**
6015632	Europe	Finland	Porvoo	*Betula pendula* (on *Inonotus obliquus*)	Hap3	**OP295158**	**OP295285**	**OP352362**	**OP352423**
6040724	Europe	Finland	Rovaniemi	*Picea* sp.	Hap3	**OP295159**	**OP295286**	**OP352363**	**OP352424**
6054622	Europe	Finland	Raahe	*Betula* sp.	Hap3	**OP295160**	**OP295287**	**OP352364**	**OP352425**
6054716	Europe	Finland	Utsjoki	*Betula* sp.	Hap2	**OP295161**	**OP295288**	**OP352365**	**OP352426**
BRNM 705020	Europe	Hungary	Szabolcs-Szatmár-Bereg	*Quercus robur*	Hap3	**OP295162**	**OP295289**	**OP352366**	**OP352427**
64251	Europe	Norway	Sogndal, Sogn Og Fjordane		Hap3	**OP295163**	**OP295290**	MG593281	**OP352428**
65268	Europe	Norway	Eidsvoll, Akershus		Hap3	**OP295164**	**OP295291**	**OP352367**	**OP352429**
68241	Europe	Norway	Oppegård, Akershus		Hap3	**OP295165**	**OP295292**	**OP352368**	**OP352430**
69367	Europe	Norway	Nesodden, Akershus		Hap3	**OP295166**	**OP295293**	**OP352369**	**OP352431**
69689	Europe	Norway	Alta, Finnmark		Hap3	**OP295167**	**OP295294**	**OP352370**	**OP352432**
220192	Europe	Norway	Tvedestrand, Aust-Agder		Hap3	**OP295168**	**OP295295**	**OP352371**	**OP352433**
230773	Europe	Norway	Trondheim, Sør-Trøndelag		Hap3	**OP295169**	**OP295296**	**OP352372**	**OP352434**
284607	Europe	Norway	Rygge, Østfold		Hap3	**OP295170**	**OP295297**	**OP352373**	**OP352435**
286068	Europe	Norway	Eidsvoll, Akershus		Hap3	**OP295171**	**OP295298**	**OP352374**	**OP352436**
286284	Europe	Norway	Kongsvinger, Hedmark		Hap3	**OP295172**	**OP295299**	**OP352375**	**OP352437**
291654	Europe	Norway	Målselv, Troms		Hap3	**OP295173**	**OP295300**	**OP352376**	**OP352438**
295520	Europe	Norway	Storfjord, Troms		Hap3	**OP295174**	**OP295301**	**OP352377**	**OP352439**
7026019	Europe	Poland	Białowieża	*Carpinus betulus*	Hap3	**OP295175**	**OP295302**	**OP352378**	**OP352440**
7029323	Europe	Russia	Taldom	*Betula* sp.	Hap3	**OP295147**	**OP295274**	**OP352352**	**OP352412**
Kout 3	Europe	Russia	Karelia	*Betula* sp.	Hap3	**OP295148**	**OP295275**	**OP352353**	**OP352413**
BRNM 709971	Europe	Slovakia	Pezinok	*Alnus glutinosa*	Hap3	**OP295176**	**OP295303**	**OP352379**	**OP352441**
80180	Europe	UK	Windsor Great Park		Hap3	**OP295177**	**OP295304**	**OP352380**	**OP352442**
CBS 446.50	North America	Canada	British Columbia		Hap4	**OP295178**	**OP295305**	**OP352381**	**OP352443**
CBS 357.34	North America	USA			Hap13	MH855565	MH867070		
DL96-261	North America	USA	Michigan	Hardwood	Hap4	**OP295179**	**OP295306**	**OP352382**	**OP352444**
DL96-262	North America	USA	Michigan	Hardwood	Hap4	**OP295180**	**OP295307**	**OP352383**	**OP352445**
DL96-574	North America	USA	Michigan	Hardwood	Hap4	**OP295181**	**OP295308**	**OP352384**	**OP352446**
DLC97-166	North America	USA	Wisconsin	*Populus* sp.	Hap4	**OP295182**	**OP295309**	**OP352385**	**OP352447**
FP-102050	North America	USA	Alaska	*Betula* sp.	Hap5	**OP295183**	**OP295310**	**OP352386**	**OP352448**
FP-102250-Sp	North America	USA	Wisconsin	*Thuja* sp.	Hap4	**OP295184**	**OP295311**	**OP352387**	**OP352449**
FP-102318-Sp	North America	USA	Wisconsin		Hap4	**OP295185**	**OP295312**	**OP352388**	**OP352450**
FP-105267-Sp	North America	USA	Maryland		Hap4	**OP295186**	**OP295313**	**OP352389**	**OP352451**
FP-106899-Sp	North America	USA	Mississippi		Hap4	**OP295187**	**OP295314**	**OP352390**	**OP352452**
FP-134973-Sp	North America	USA	New York	*Ulmus* sp.	Hap4	**OP295188**	**OP295315**	**OP352391**	**OP352453**
FP-151129	North America	USA	Michigan	*Abies* sp.	Hap11	**OP295189**	**OP295316**	KP134866	**OP352454**
GAL-3333	North America	USA	Alaska		Hap5	**OP295190**	**OP295317**	**OP352392**	**OP352455**
HHB-15056	North America	USA	Alaska		Hap5	**OP295191**	**OP295318**		**OP352456**
HHB-15239	North America	USA	Alaska	*Betula papyrifera*	Hap4	**OP295192**	**OP295319**	**OP352393**	**OP352457**
HHB-17181	North America	USA	Virginia	Hardwood	Hap4	MG572753	MG572737	MG593282	**OP352458**
HHB-18747	North America	USA	Illinois	*Liriodendron tulipifera*	Hap12	**OP295193**	**OP295320**	**OP352394**	**OP352459**
N.L. Bougher NLB 1155	Oceania	Australia	Perth		Hap9	MT537000	MT524535		
916456	Oceania	New Zealand	Southland		Hap10	**OP295194**	**OP295321**	**OP352395**	**OP352460**
ICMP16418	Oceania	New Zealand	Stewart Island		Hap10	**OP295195**	**OP295322**		**OP352461**
PDD68418	Oceania	New Zealand	Three Kings Islands		Hap10	**OP295196**	**OP295323**		**OP352462**
18.MAR.02	South America	Argentina			Hap14	**OP295197**	**OP295324**		**OP352463**
N.11901	South America	Argentina	Neuquén		Hap14	**OP295198**	**OP295325**	**OP352396**	**OP352464**

Accessions generated in this study are bolded.

### Haplotype TCS network

**Fig 3:**
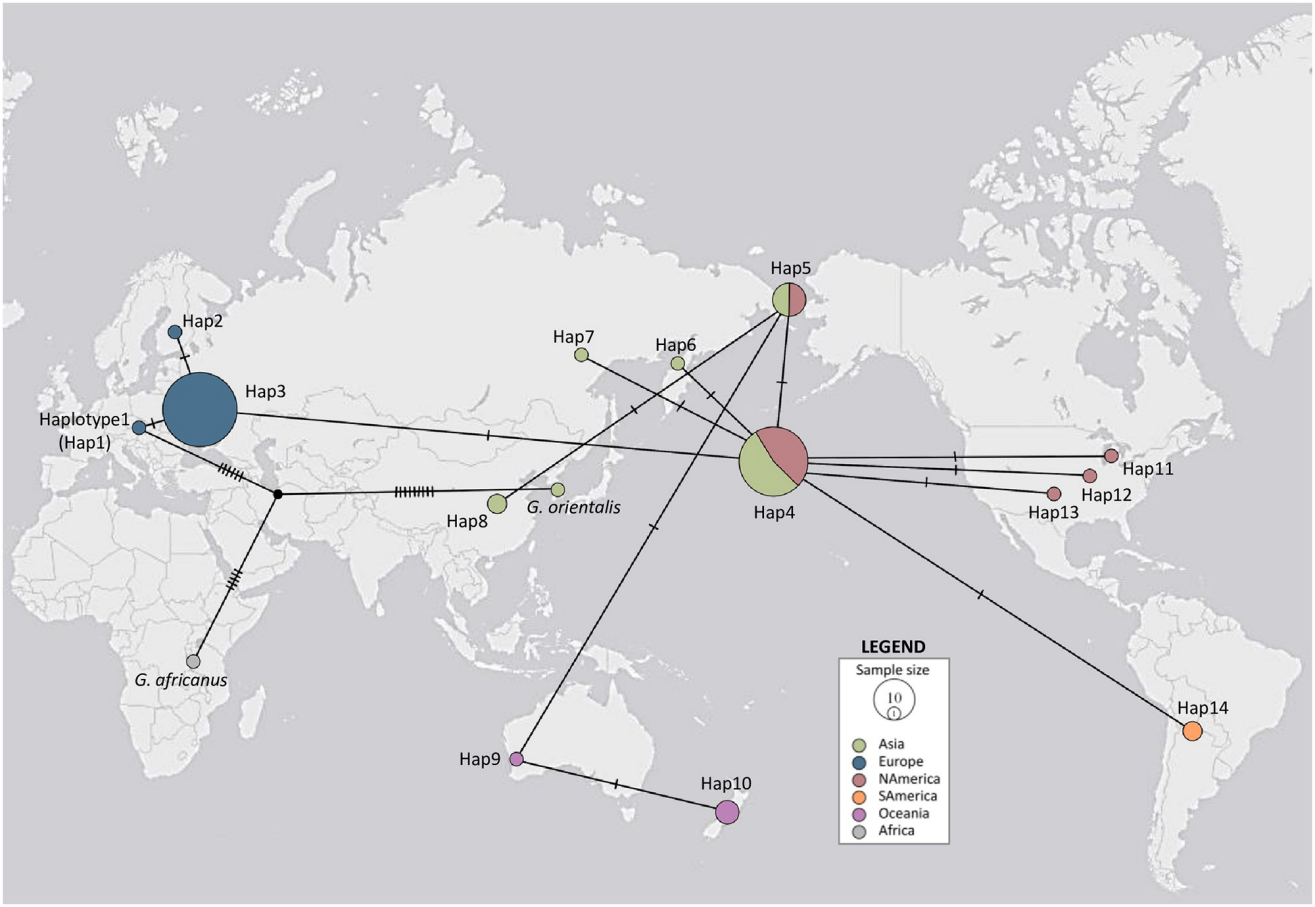
TCS haplotype networks of *Gloeoporus dichrous* specimens on the world map. Haplotypes were constructed based on ITS + nrLSU + *rpb2* + *tef1* dataset. Each colored circle represents a haplotype, while a black circle illustrates a theoretical haplotype. Each color represents a continent, as indicated in the legend, and the number of hatch marks on network branches specifies the number of mutations. The size of the circle is proportional to the frequency of each haplotype. World map credit: USGS National Map Viewer (http://viewer.nationalmap.gov/viewer/); modified for an illustrative purpose only.

The haplotype network results were analogous to the results of the divergence time analyses, where the haplotypes of *G*. *dichrous* specimens primarily corresponded with biogeographical locations (Fig 3). Generally, there is a clear division of haplotypes between continents. Haplotypes consisting of Asian and North American specimens (Hap4 and Hap5) served as the center of divergence. Hap4, with specimens from Eastern Asia and North America, was the core of all haplotypes, as the haplotypes of other Asian (Hap6 and Hap7), North American (Hap11, Hap12, and Hap13), and South American (Hap14) specimens diverged from Hap4. Hap5 consisted of specimens from two distant regions: Eastern Asia (China, the Republic of Korea, and Taiwan) and North America (USA) (see Table 1). Specimens from Oceania diverged from Hap5 and were divided into two haplotypes: Hap9 for a specimen from Australia and Hap10 for specimens from New Zealand.

Some haplotypes consisting of a single specimen were separated from the core continental haplotypes. Hap11, Hap12, and Hap13, each comprising a single specimen from the USA, were distinct from Hap4 and Hap5 with the remaining 15 specimens from North America. Similarly, Hap1 and Hap2, each consisting of a specimen from the Czech Republic and Finland, respectively, diverged from the core Hap3 with the rest of the 27 European specimens. A theoretical haplotype for the last common ancestor (LCA) of *G*. *africanus*, *G*. *dichrous*, and *G*. *orientalis* was estimated between the haplotypes of *G*. *africanus*, *G*. *orientalis*, and Hap1 with a specimen from the Czech Republic.

## Discussion

Analyses of datasets through divergence time, biogeographic distribution, and haplotypes supported the divisions of *G*. *dichrous* specimens into five continents: Asia, Europe, North and South America, and Oceania.

### Divergence time and biogeographic diversification

The chronogram for Polyporales supported the phylogenetic relationship between each genus and species; however, some sequences were not grouped within the identified species clade. *Gloeoporus dichrous* specimens F10240 (Taiwan), HHB-15056 (USA), and 18.MAR.02 (Argentina) did not belong to the *Gloeoporus dichrous* clade (Fig 1). In addition, *Gloeoporus guerreroanus* ICN 139059 grouped monophyletically with *Meruliopsis cystidiata* 776308 in the *Meruliopsis* clade, with high support for the genus (posterior probability/bootstrap = 1/98, S1 Fig). This aligns with a previous report on *M*. *cystidiata* that recognized *G*. *guerroroanus* as conspecific [19]. Validating the morphological characteristics of the specimens of these sequences may ensure that they are placed in an appropriate clade.

Based on the divergence time and biogeographic diversification analyses, the origin of *G*. *dichrous* was estimated to be either Asia or North America (Fig 2). Since their origin, different populations have diverged and dispersed to other parts of the world. Considering that speciation occurred between 6.4 and 3.1 MYA for other WDF [17, 20, 21], *G*. *dichrous* populations unprecedentedly avoided vicariant speciation, despite the widespread distribution and varying environmental conditions for over 10 million years (Fig 1). Therefore, regardless of population divisions, the morphological characteristics of *G*. *dichrous* are relatively constant [22–25]. These include a gelatinous pore surface that changes color with age from reddish to purplish-brown, and these characteristics are different from those of other closely related species, such as *G*. *africanus* and *G*. *orientalis* [26].

European populations were separated into two clades (Figs 1 and 2). One clade consisted of populations from Northern Europe and the other from Central Europe. Divisions within the European specimens may be explained by the segmentation of terrestrial biomes over the Pleistocene glaciation, which aligns with the divergence period (approximately 2.0 MYA) of the two European clades (Fig 1). Northern Europe contains boreal forests, whereas Central Europe contains temperate forests (S2 Fig) [27]. Differences in the biomes were also partially reflected in the tree hosts of *G*. *dichrous* (Table 1). Coniferous evergreen trees commonly found in boreal forests such as spruce (*Picea*) have been recorded as host trees in Northern Europe, whereas a wide variety of trees such as alder (*Alnus*), fir (*Abies*), poplar (*Populus*), and oak (*Quercus*) have been recorded as hosts in the temperate deciduous forests of Central Europe. For the other continental populations, most specimens from the USA were grouped within the North American clade, but some from western North America were grouped in the “Pacific” clade, together with specimens from Asia (Fig 1). Specimens from this “Pacific” clade may have dispersed through the Bering Land Bridge (BLB) approximately 5.5 to 5.4 MYA [28]. The BLB has been speculated to have served as the transfer route for other fungi, such as *Boletus*, *Bondarzewia*, and *Hydnum* [21, 29, 30]. The “Pacific” lineage of *G*. *dichrous* that is difficult to explain by continental distribution serves as evidence of some unconstrained movements of the species.

### Haplotype network

The TCS haplotype network largely corresponded to the results of the divergence time and biogeographical diversification analyses. Based on each haplotype association, *G*. *dichrous* diverged from the LCA of *G*. *africanus* and *G*. *orientalis*, with many accumulated nucleotide mutations (n = 6 for the ITS + nrLSU + *rpb2* + *tef1* dataset; Fig 3). Overall, despite the general division of populations by continent, the haplotype groups were not isolated, indicating continuous gene flow between the populations.

*Gloeoporus dichrous* from Europe showed relatively low genetic diversity, except for a few discrete populations (Hap1 and Hap2; Fig 3). However, the discrete populations did not have a distinct character (host identity or country location) from that of Hap3, the main European haplotype (Table 1). This implies that the haplotypes within Europe are relatively stable and that the impact of disparate biomes on haplotype patterns is smaller than that on nucleotide changes. For the Asian groups, the complex network of Hap4, Hap5, and Hap8 demonstrates how diverse populations move within the continent. The North American groups in Hap4 and Hap5, in addition to the haplotypes from inland North America (Hap11, Hap12, and Hap13), also show how populations have moved freely within a continent. Hap4 and Hap5, with specimens spanning a large continental area from Asia to Alaska in North America, may explain the dispersal routes to and from Asia to North America. The specimens in Hap4 and Hap5 were mostly found in the temperate regions of the Northern Hemisphere (S2 Fig) on diverse tree species (Table 1). These similar biomes may have facilitated the stability of *G*. *dichrous* haplotypes.

Hap9 of an Australian specimen was closely related to Hap5 of specimens from Asia and Alaska, USA (Fig 3). The *Gloeoporus dichrous* population from southeastern Asia could have been transferred across Wallace’s Line to Australia. Several wind- and human-mediated dispersion mechanisms for biological species across Wallace’s Line have been suggested [31–33], and these are applicable to *G*. *dichrous*. The lightweight, spore-bearing, and plant-mediated characteristics of *G*. *dichrous* may have facilitated its long-distance dispersal. New Zealand became isolated from Gondwanaland approximately 84 MYA [34] and has sustained much of the island’s endemic biological diversity [35]. However, it has become prone to invasive species because of several factors such as climate change and human activities [36, 37]. Thus, the New Zealand *G*. *dichrous* population (Hap10) could have been derived from Australian populations and settled as a discrete population.

### Dispersal mechanisms

Several mechanisms have been suggested for fungal dispersal, including long-distance spore dispersal and animal dispersal [38, 39]. The growth of mycelia in host plants also explains how plant immigration facilitates the long-distance dispersal of associated fungi [17, 21]. The diverse host residences of *G*. *dichrous* may have eased the spread of mycelia and basidiospores across continents, enhancing their survival. *Gloeoporus dichrous* grows on various dead or living trees, including angiosperms, such as *Betula* and *Quercus* [23], and gymnosperms, such as *Picea* and *Pinus* (Table 1). The widespread dispersion of diverse tree species during the Neogene period may have enabled the transfer of *G*. *dichrous* [40, 41]. Motile organisms, such as insects [42], may also be possible dispersal vectors. Insect vectors could have carried pieces of *G*. *dichrous* mycelia or basidiospores to many different types of host trees and even allowed their development on dead basidiocarps of other hymenochaetoid polypores, such as *Inonotus obliquus* [43] (Table 1).

Continents such as South America are far less explored than other regions to fully evaluate a species distribution worldwide [44], which leaves uncertainty in discovering the prime contributors that drive the global distribution of each ecological or taxonomic group of fungi. For *G*. *dichrous*, the species has only been reported from Morocco within Africa, without sequence data [45]. Therefore, additional sampling and molecular assessment of *Gloeoporus* species and their relatives in Africa and South America are required to expand the scope of this study. In addition, the small number of specimens studied impeded determination of the precise origin of *G*. *dichrous*. Collecting and assessing additional *Gloeoporus* specimens will allow us to estimate the crown age more accurately, and expansion of the number of genetic markers used in distribution analyses may reveal more populations and convincing dispersion routes.

## Conclusion

The cosmopolitan wood decay species *Gloeoporus dichrous* was analyzed using multi-marker data by Bayesian inference-based phylogenetic analysis to predict molecular dating and visualize the phylogeography. Similar to other WDF, this species has mainly been divided into biogeographical populations by continent since 10.3 MYA (median time). Numerous possible mechanisms may explain the dispersion of *G*. *dichrous*, including the transfer of mycelia and basidiospores by the wind or host. The varying times and introduction routes of *G*. *dichrous* to each continent were also predicted. The distribution pattern of *G*. *dichrous* analyzed in this study may contribute to a broader picture of polypore dispersion and speciation.

## Materials and methods

### DNA sequencing

Genomic DNA was extracted from small hymenophore pieces of *Gloeoporus dichrous* specimens collected from diverse continents using a modified CTAB extraction protocol [46]. Four different genetic regions were amplified by PCR—ITS, nrLSU, *rpb2*, *tef1*—using the AccuPower PCR premix (Bioneer, Daejeon, Korea). Primers ITS1F / ITS4B [47] were used to amplify ITS, LR0R / LR5 [48] for nrLSU, RPB2-6F1 / bRPB2-7.1R [49] for *rpb2*, and EF595F / EF1160R [50] for *tef1*. The PCR were performed using a C100 thermal cycler (Bio-Rad, USA) with the following conditions for ITS, nrLSU, and *tef1*: 95°C for 5 min; 35 cycles of 95°C for 40 s, 55°C for 40 s, and 72°C for 1 min; and lastly 72°C for 10 min. The PCR conditions for *rpb2* were as follows: 95°C for 5 min; 35 cycles of 95°C for 1 min, 50°C for 1 min, a ramp of 0.3°C per second to 72°C, 72°C for 1 min; and lastly 72°C for 10 min.

The PCR products were electrophoresed on a 1% agarose gel to verify the PCR and then purified using an Expin^™^ PCR Purification Kit (GeneAll Biotechnology, Seoul, Korea). DNA sequencing was performed using the PCR primers on an ABI Prism 3700 Genetic Analyzer (Life Technologies, USA) at Macrogen (Seoul, Korea). All sequences were proofread and edited using Geneious Prime 2022.0.2 software (www.geneious.com). Additional *G*. *dichrous* sequences of the four genetic regions were retrieved from NCBI GenBank.

### Molecular dating and phylogeography

A total of 77 *Gloeoporus dichrous* strains with sequences for at least two genetic regions were analyzed (Table 1). The sequences were assembled for each genetic region and aligned using MAFFT version 7 software [51] with the default settings. Manual trimming was performed at the ends of the alignment. The sequences of the four genetic regions were concatenated with the following partitions: ITS 1,397 bases, nrLSU 905 bases, *rpb2* 903 bases, *tef1* exon 1 388 bases, *tef1* intron 131 bases, and *tef1* exon 2 1,174 bases. For the phylogenetic analyses, the partition model was independently selected for each partition by bModelTest [52]. The initial trees were constructed using RAxML 8.2.12 software [53] using concatenated sequences with branches re-rooted with outgroup sequences (S1 Table). The trees were converted into an ultrametric tree using the convert_to_ultrametric function of ete3 3.1.2 module [54].

Bayesian evolutionary analysis was conducted for the concatenated *G*. *dichrous* sequences using BEAST 2.6.7. software [55]. Optimised relaxed clock (ORC) model with estimated rates and birth-death model speciation priors was used to estimate the divergence time. In total, 500 million MCMC analyses were performed for chain convergence, with scaleFactors adjusted according to six rounds of 100 million MCMC analyses using the BEAST2 output log. ESS values over 200 of chain convergence were verified using Tracer v1.7.2 software (http://tree.bio.ed.ac.uk/software/tracer/). Molecular dating of *G*. *dichrous* was based on the fossil priors of Agaricomycetes (estimated to have diverged between 372 and 222 MYA) [56], Agaricales (94–90 MYA) [57], and Hymenochaetales (118–113 MYA) [58], employed by a Clade Age model [59]. After the BEAST2 analysis, 10% of trees were removed by burnin using a Logcombiner, and summarization was performed using the Treeannotator of BEAST 2.6.7. The resulting tree across geological ages was visualized with the 95% highest posterior density (HPD) range. A geologic timeline was supplemented using the geoscalePhylo function of the strap 1.6.0 module with R 4.1.2. [60]. Subtrees of the RAxML analysis and BEAST2 were compared, and the bootstrap values of common subtrees were mapped onto the resulting tree using the ape 5.6.2 [61] and geiger 2.0.10 modules [62].

The ancestral location of *G*. *dichrous* was estimated using the statistical-dispersal-extinction-cladogenesis (S-DEC) model in RASP 4.0 [63]. The posterior distributions of the ITS + nrLSU + *rpb2* + *tef1* multi-marker phylogeny from BEAST were used for analysis. The geographical areas were divided by continent.

### Haplotype analysis

Different populations of *G*. *dichrous* were predicted through haplotype analysis using the same four genetic regions as used for the molecular dating. The three specimens (F10240, 18.MAR.02, and HHB-15056) that were excluded from the *G*. *dichrous* clade in the phylogenetic tree were retained in the analysis. The sequences of *G*. *africanus* and *G*. *orientalis*, sister species of *G*. *dichrous*, were also included in the assessment to estimate the ancestral haplotype of *G*. *dichrous*. The haplotypes were constructed using PopART [64] with TCS algorithm [65]. The locations (traits) of the specimens were labeled by continent. The network was placed on a world map with each haplotype placed approximately near the location where most of the specimens were collected. Haplotype group for each specimen is listed in Table 1.

## Supporting information

S1 Fig Chronogram for *Gloeoporus dichrous* based on ITS + nrLSU + *rpb2* + *tef1* dataset, constructed using BEAST CladeAge. A geologic timeline and node bars for the highest-posterior-density interval containing 95% of the posterior distribution are displayed.
(PDF)

S2 Fig Locations of *Gloeoporus dichrous* specimens in Europe and haplotype Hap4 based on ITS + nrLSU + *rpb2* + *tef1* dataset. North Europe specimen localities are indicated by orange, Central Europe specimens by yellow, and Hap4 specimens by black location icons. Temperate regions are presented in green and coniferous regions are presented in blue. World map credit: NASA Earth Observatory (https://earthobservatory.nasa.gov/biome); modified for an illustrative purpose only.
(PDF)

S1 Table GenBank descriptions and accession numbers for the outgroup species assessed in this study. (XLSX)

S1 File Aligned concatenated (ITS + nrLSU + *rpb2* + *tef1*) sequences of all analyzed sequences in this study. (FASTA)
