# Supplementary figures and images for: Global phylogeographical distribution of Gloeoporus dichrous
Source: PLoS One. 2023 Jul 13;18(7):e0288498. doi: 10.1371/journal.pone.0288498 (PMC10343081; doi:10.1371/journal.pone.0288498)

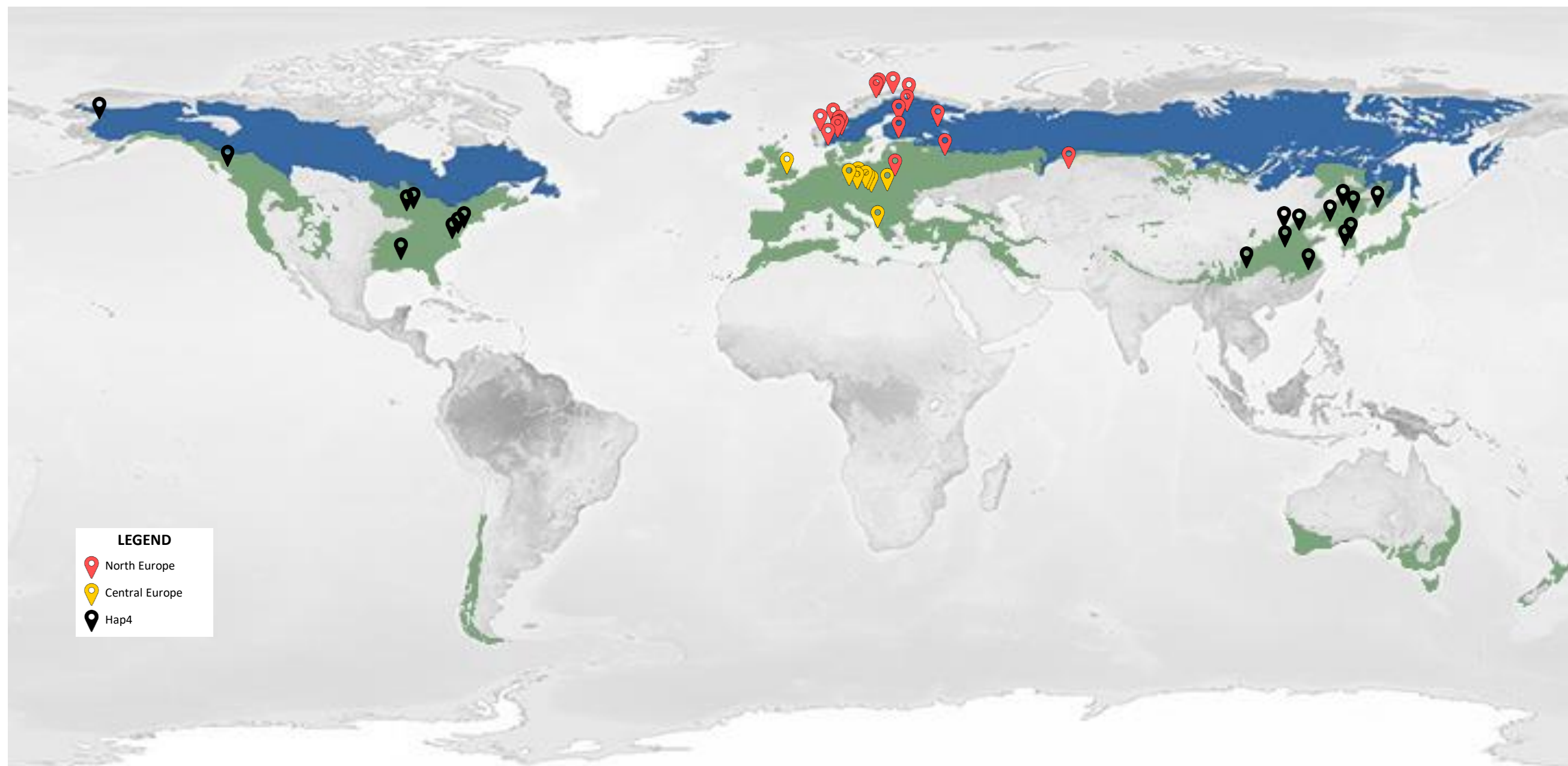

Supplement: S2 Fig — North Europe specimen localities are indicated by orange, Central Europe specimens by yellow, and Hap4 specimens by black location icons. Temperate regions are presented in green and coniferous regions are presented in blue. World map credit: NASA Earth Observatory (https://earthobservatory.nasa.gov/biome); modified for an illustrative purpose only. (PDF) [file pone.0288498.s002.pdf]
