# Supplementary material for: Global phylogeographical distribution of Gloeoporus dichrous
Source: PLoS One. 2023 Jul 13;18(7):e0288498. doi: 10.1371/journal.pone.0288498 (PMC10343081; doi:10.1371/journal.pone.0288498)
Supplement: S1 Fig — A geologic timeline and node bars for the highest-posterior-density interval containing 95% of the posterior distribution are displayed. (PDF) [file pone.0288498.s001.pdf]

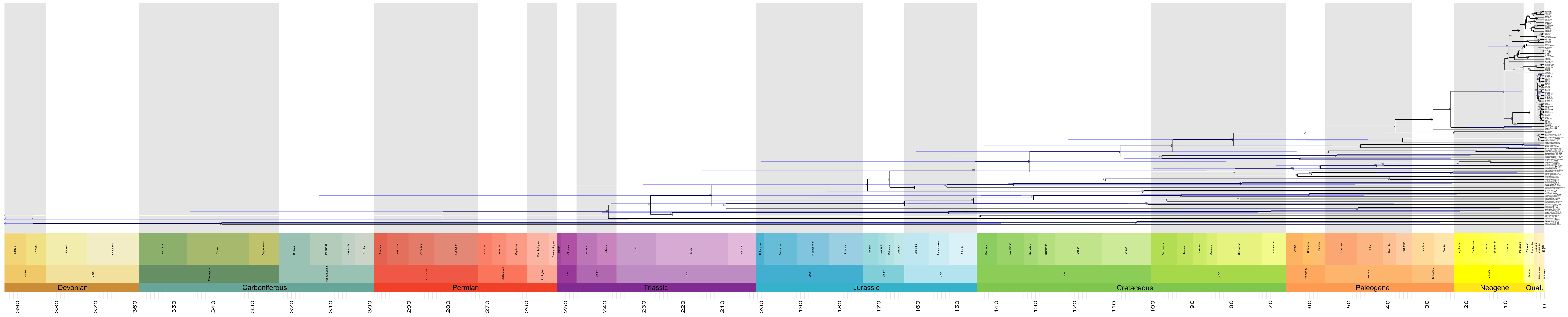
Devonian
Carboniferous
Permian
Triassic
Jurassic
Cretaceous
Paleogene
Neogene
Quat.
390
380
370
360
350
340
330
320
310
300
290
280
270
260
250
240
230
220
210
200
190
180
170
160
150
140
130
120
110
100
90
80
70
60
50
40
30
20
10
0
